# Prophylactic Antibiotics in Hip Fracture Surgery: A Randomized Prospective Study

**DOI:** 10.7759/cureus.46460

**Published:** 2023-10-04

**Authors:** Syed Kamran Ahmed, Tauqeer Khan, Salman Javed, Ibrahim Zahid Selod, Shakeel Gul Shaikh

**Affiliations:** 1 Orthopedics, The Indus Hospital, Karachi, PAK; 2 Orthopedics, United Medical and Dental College, Karachi, PAK

**Keywords:** morbidity, antibiotics, surgical site infection, prophylaxis, hip surgery

## Abstract

Introduction

Hip fracture surgeries constitute a large portion of orthopedic surgeries, and infective complications are one of the most severe and devastating sequels following fixations or replacements. Preoperative antibiotic prophylaxis is an important means to control SSI. Thus, we set out to assess the impact of a single dose versus three doses of antibiotics on surgical site infections in patients undergoing hip surgery.

Materials and methods

A randomized controlled trial was conducted at the Department of Orthopaedics, The Indus Hospital, Karachi, Pakistan. All patients admitted for hip fracture surgery who met the inclusion criteria were enrolled and divided into two groups. One group (Group A) was given a single dose of antibiotics preoperatively, and the other (Group B) was given three doses, one preoperatively and two postoperatively. Patients were assessed for wound condition and signs of infection. Data were entered and analyzed using SPSS version 21.0. The chi-square test was applied to assess the significant association between both the groups and SSI. A significant statistical association was noted when the P value was found to be <0.05.

Results

The study included 62 patients, with the majority of them being females (n=33; 53.2%). The mean age of the patients was 60.5±15.1 years. Only three (4.8%) patients developed SSI. No statistically significant association was detected between surgical site infections and the two antibiotic regimens being administered after controlling for the confounders.

Conclusion

There was no statistical relationship between surgical site infections with a single dose versus three doses of antibiotics in patients undergoing hip surgery.

## Introduction

Surgical site infection (SSI) is defined as microbial contamination at the surgery site within 30 days of surgery; however, in orthopedic cases where an implant is placed, the time extends to one year after the placement of an implant in the body [1]. SSI in surgery and particularly in orthopedic patients is not only extremely devastating but at times difficult to manage [2]. Prolonged hospital stay, morbidity, disability, increased economic burden, and increased mortality are complications of SSI [3].

Although such complications are a problem for any SSI patient; however, their impact on hip fracture patients is extremely severe [4,5]. Hence, it is important to prevent SSI using various strategies to prevent morbidity and mortality. Maintaining sterilization, appropriate scrubbing, prophylactic administration of antibiotics at the proper time and that too of correct strength, reducing staff flow in the operating room, postoperative wound care, and controlling patient comorbidity can reduce SSI significantly [2,6].

The role of antibiotic prophylaxis is paramount in preventing SSI. It is crucial to ensure an adequate antibiotic concentration about 15 to 120 minutes before surgical incision to have good outcomes [7]. Apart from the timing, the choice of antibiotics and duration of antibiotics should also be considered. As far as the choice of antibiotics is concerned, the antibiotic used should be inexpensive, nontoxic, but it should cover the most prevalent organisms in prosthetic-related infections (i.e., Gram-positive Staphylococcus aureus and epidermidis) [8].

The duration of prophylactic antibiotics has been a much-debated topic, with studies recommending different results, ranging from three to five doses or even 14 days [5,7,9]. Musmar et al. suggest that antibiotics should be discontinued within 24 h after the end of surgery to prevent the emergence of resistance [9]. While, on the other hand, Thonse et al. recommended a prophylactic antibiotic regimen at the time of induction of anesthesia and two subsequent doses at 8 and 16 h postoperatively [10]. Similarly, Andersson et al. suggest the same recommendations of three doses within 24 h [11].

Despite the established benefit of prophylactic antibiotics to prevent SSI, on a literature search, no single similar study was available in our Pakistani population. Therefore, this provided a strong rationale to conduct a study and thus be able to recommend the duration of antibiotics which would not only prevent surgical site infection but would also prevent antibiotic resistance.

## Materials and methods

A pilot randomized controlled trial was conducted at the Department of Orthopaedics, The Indus Hospital, Karachi, Pakistan. This study was approved by the Institutional Review Board (Interactive Research and Development), and the study was also registered at www.ird.global (IRD_IRB_2019_07_004). The sample size was calculated using open epi software (http://www.openepi.com/SampleSize/SSCohort.htm), which was 792 (396 per arm). As this was a large sample size, and to determine the safety of the trial, a pilot study was conducted, enrolling a total of 62 patients (i.e., 31 patients per arm), which could provide a basis for our future direction. Non-probability consecutive sampling was used. The study included patients aged between 18 to 80 years of either gender, undergoing surgery for hip fractures, which included total hip arthroplasty, hemiarthroplasty, dynamic hip screw, dynamic condylar screws, proximal femur nails, and cannulated hip screws. Those patients having open fractures, taking steroids (through history), pregnant females (positive B-hcg), or were already on antibiotics for some other infection were excluded from the study.

Before the beginning of the surgery, informed consent was obtained from all the patients who were then put into one of the study arms after randomization. The primary investigator opened the sealed envelopes provided by the Indus Hospital Research Center’s Clinical Research Unit (CRU) that provided the study arm allocation. The envelopes followed the SNOSE protocol (i.e., they were sequentially numbered, opaque sealed envelopes). Before opening the envelope, the primary investigator wrote the patient’s medical record number and date and signed the envelope. The envelope contained carbon paper which transferred the data allocation paper inside. 

One group (Group A) was given a single dose of antibiotics preoperatively (i.e., injection of cefuroxime 1.5 gm, and the other (Group B) was given three doses, one preoperatively and two postoperatively). The patient underwent careful wound assessment which was done on the third, 14th, and 28th postoperative days, and the findings related to wound condition and signs of infection (erythema, tenderness, discharge, temperature change), if any, were recorded on a predesigned questionnaire by the primary investigator.

Data was entered and analyzed using SPSS 21. Mean (SD) was computed for age, incision length, and duration of surgery. Frequency and percentage were computed for gender, comorbid, and primary diagnosis SSI developed. Effect modifiers were controlled through stratification of age, gender, incision length, duration of surgery, primary diagnosis, and comorbid (hypertension, diabetes, chronic renal disease). A chi-square test was applied to assess the significant association between both the groups and SSI. P-value <0.05 was considered statistically significant.

## Results

A total of 62 patients were enrolled in the study with 31 (50%) patients in each intervention group. More than half of the patients (n=33; 53.2%) were females. The mean (±SD) age of all the patients was 60.5 (±15.1) years. The median (IQR) length of incision of all the patients was 11 (10-12). The median duration of surgery was 80 (60-90) minutes. Half of the patients, 31 (50%), underwent surgery for intertrochanteric femur fractures (underwent dynamic hip screw) followed by the neck of femur fractures 28 (45.2%) out of which 20 patients underwent hemiarthroplasty, and eight had a total hip replacement. All patients with subtrochanteric femur fractures underwent proximal femoral nails. In terms of comorbidities, hypertension was the most prevalent among the study participants, with 27 (43.5%) of the participants suffering from it, followed by diabetes mellitus 20 (32.3%). In terms of surgical site infection, the majority of the patients 59 (95.2%) did not develop surgical site infections, with only three (4.8%) patients developing SSI (Table 1).

**Table 1:** Stratified Analysis ƚ: Fisher's exact test P value <0.05 was considered statistically significant. n: number of participants %: percentage of the participants

Variables		Surgical site Infection		P value
		Absent	Present	
		n (%)	n (%)	
Gender				
Female	Group A	19 (61.3%)	01 (50%)	1.00^ƚ^
	Group B	12 (38.7%)	01 (50%)	
Male	Group A	10 (35.7%)	01 (100%)	0.37^ƚ^
	Group B	18 (64.3%)	0 (0.0%)	
Primary Diagnosis				
Neck of Femur Fractures	Group A	13 (48.2%)	01 (100%)	1.00^ƚ^
	Group B	14 (51.9%)	0 (0.0%)	
Intertrochanteric Femur Fractures	Group A	14 (48.3%)	01 (50%)	1.00^ƚ^
	Group B	15 (51.7%)	01 (50%)	
Age				
≤64 Years	Group A	15 (50%)	0 (0.0%)	1.00^ƚ^
	Group B	15 (50%)	1 (100%)	
>64 Years	Group A	14 (48.3%)	02 (100%)	0.48^ƚ^
	Group B	15 (51.7%)	0 (0.0%)	
Hypertension				
No	Group A	15 (44.1%)	01 (100%)	0.46^ƚ^
	Group B	19 (55.9%)	0 (0.0%)	
Yes	Group A	14 (56%)	01 (50%)	1.00^ƚ^
	Group B	11 (44%)	01 (50%)	
Diabetes Mellitus				
Yes	Group A	18 (43.9%)	01 (100%)	0.45^ƚ^
	Group B	23 (56.1%)	0 (0.0%)	
No	Group A	11 (61.1%)	01 (50%)	1.00^ƚ^
	Group B	07 (38.9%)	01 (50%)	
Incision Length				
≤11 cm	Group A	21 (58.3%)	02 (100%)	0.5^ƚ^
	Group B	15 (41.7%)	0 (0.0%)	
>11 cm	Group A	08 (34.7%)	0 (0.0%)	1.00^ƚ^
	Group B	15 (65.2%)	01 (100%)	
Duration of Surgery				
≤80 cm	Group A	15 (46.8%)	01 (100%)	0.48^ƚ^
	Group B	17 (53.1%)	0 (0.0%)	
>80 cm	Group A	14 (51.8%)	01 (50%)	1.00^ƚ^
	Group B	13 (48.2%)	01 (50%)	

The baseline characteristics of the two intervention groups (i.e., group A and group B) were compared using a t-test and Mann-Whitney U test for quantitative variables. For categorical variables, the chi-square and Fisher's exact test were used. The only statistically significant difference was detected between the incision length of the two intervention groups with group B having an incision length longer than group A (p=0.05) (Table 2).

**Table 2:** Patient Demographics n: number of participants %: percentage of the participants SD: standard deviation

Demographical information of patients n=62	
Age	
Mean ± SD	60.5±15.1
Min-Max	25 – 84
Gender	
Male	29 (46.7%)
Female	33 (53.2%)
Primary Diagnosis	
Neck of Femur Fractures	28 (45.2%)
Intertrochanteric Femur Fractures	31 (50%)
Sub-trochanteric Femur Fractures	03 (4.8%)
Comorbid	
None	27 (43.5%)
Hypertension	27 (43.5%)
Diabetes	20 (32.3%)
Chronic Kidney Disease	01 (1.6%)
Others	01 (1.6%)
Incision Length	
Median (IQR)	11 (10-12)
Duration of Surgery (mins)	
Median (IQR)	80 (60-90)
Surgical Site Infection	
Absent	59 (95.2%)
Present	03 (4.8%)
Intervention Group	
Group A	31 (50%)
Group B	31 (50%)

No statistically significant association was detected between the surgical site infections and the two antibiotic regimens being administered (i.e., group A and group B after controlling for the confounders, that is, age, gender, incision length, duration of surgery, primary diagnosis, and comorbidities (hypertension and diabetes)) (Table 3).

**Table 3:** Patient Demographics Among Intervention Groups Ŧ: T-test, ¶: Mann-Whitney U test, §: Chi-Square, ƚ: Fisher's exact test P-value <0.05 is considered statistically significant. N: number of participants %: percentage of the participants

Variable	Group A N (%)	Group B N (%)	P value
Surgical Site Infection			
Absent	29 (93.5%)	30 (96.8%)	1.00^ƚ^
Present	02 (6.5%)	01 (3.2%)	
Age	61.1 ± 15	59.9 ± 15.3	0.76^Ŧ^
Gender			
Male	11 (35.5%)	18 (58.1%)	0.07^§^
Female	20 (64.5%)	13 (41.9%)	
Duration of Surgery Median (IQR)	80 (65-90)	80 (55-90)	0.45^¶^
Incision Length Median (IQR)	10 (08-12)	12 (10-12)	0.05^¶^
Primary Diagnosis			
Neck of Femur Fractures	14 (45.2%)	14 (45.2%)	0.83^ƚ^
Intertrochanteric Femur Fractures	15 (48.4%)	16 (51.6%)	
Subtrochanteric Femur Fractures	02 (6.5%)	01 (3.2%)	
Comorbid			
None	12 (38.7%)	15 (48.4%)	0.61^§^
Hypertension	15 (48.4%)	12 (38.7%)	0.61^§^
Diabetes	12 (38.7%)	08 (25.8%)	0.42^§^
Chronic Kidney Disease	0 (0.0%)	1 (3.2%)	1.000^ ƚ^
Others	01 (3.2%)	0 (0.0%)	1.000^ƚ^

## Discussion

Our study revealed that there is no difference associated with the use of a single dose compared to three doses of antibiotics for patients undergoing hip fracture surgeries. This is consistent with previous studies reporting that additional postoperative doses of cefazolin offer no advantage over its single preoperative dose [12]. Staphylococcus species are the most commonly reported organism in SSI after orthopedic surgeries, which are likely to be intraoperative contamination from the skin of the patient, which can be prevented but appropriate strategies and precautions and adequate antibiotic coverage are needed. The prevalence of SSI for all orthopedic procedures is reported to be between 0.6 and 2.55% [2,13,14].

Literature supports the point that a short course of antibiotic prophylaxis is sufficient for clean cases and has comparable results to a long course of antibiotics. SSI studied in various surgical specialties by Fonseca et al. reported that a single dose of cefazolin was as effective as an antibiotic given for 24 h in preventing SSI and instead was beneficial in preventing antibiotic resistance [14].

However, because of the presence of implants in orthopedic cases, the risk of SSI becomes more significant, and taking measures to prevent SSI is paramount. Thus, even considering the antibiotic regime is important. Limited studies are available comparing short and long antibiotic regimens in implant-related cases [1,7]. Among the limited studies, one by Morrison et al. reported that SSI in surgical fixation of low-energy closed fractures was comparable in patients receiving single versus multiple doses of antibiotics [15]. Similar findings were noted by Slobogean et al. in patients undergoing surgery for closed long-bone fractures [16].

Antibiotic prophylaxis in orthopedic surgeries has been a topic of debate considering the emergency of antibiotic resistance, and as most of the data comes from the developed world, the balance between SSI and antibiotic resistance is a challenge for developing countries. A study conducted by Mohammed et al. in Southern India reported a high SSI rate of 11.6% in clean elective implant surgeries [17]. A possible explanation for the high reported rate of SSI in developing nations is a lack of financial resources, improper operative conditions, and, most importantly, ineffective infection prevention strategies. These are the reasons why recommendations from the developed world can often be ineffective when applied in developing nations, and, therefore, data from developing countries needs to be highlighted when discussing SSI in surgeries, especially in implant-related ones.

In our study, regression analysis was done to delineate the risk factors of infection, but none of the study factors were found significantly associated with the treatment regimes in patients undergoing hip fracture surgeries. There are a few limitations to this study. As it is a pilot study, the number of patients enrolled in this study is small compared to previously published studies. This resulted in fewer patients in our infected cohort. Such a finding would have affected our results; however, a study on a larger scale can help us guide further on the SSI in hip fractures and help set a uniform antibiotic regime.

## Conclusions

The above results demonstrate that an additional dose of antibiotics offers no advantage over single-dose antibiotics for patients undergoing hip fracture surgeries, and both the treatment regimens, single-dose and three-dose antibiotics, are equally effective against surgical site infection. However, this debate might not end here as the situation varies from place to place and person to person. Although a standard protocol may be set for all clean cases; however, even with the same country, the healthcare standards might not be the same. Therefore, it is highly recommended that apart from making a national or global policy on antibiotic regimes in hip fractures, every organization should consider conducting a similar study to find out what other factors might contribute to SSI in their healthcare setup, which might lead to a specific change in regime for them.
